# Role of Nitric Oxide in Shiga Toxin-2-Induced Premature Delivery of Dead Fetuses in Rats

**DOI:** 10.1371/journal.pone.0015127

**Published:** 2010-12-29

**Authors:** Juliana Burdet, Elsa Zotta, Maximiliano Cella, Ana M. Franchi, Cristina Ibarra

**Affiliations:** 1 Laboratorio de Fisiopatogenia, Departamento de Fisiología, Facultad de Medicina, Buenos Aires, Argentina; 2 CEFYBO-CONICET, Universidad de Buenos Aires, Buenos Aires, Argentina; Institut de Pharmacologie et de Biologie Structurale, France

## Abstract

Shiga toxin-producing *Escherichia coli* (STEC) infections could be one of the causes of fetal morbimortality in pregnant women. The main virulence factors of STEC are Shiga toxin type 1 and/or 2 (Stx1, Stx2). We previously reported that intraperitoneal (i.p.) injection of rats in the late stage of pregnancy with culture supernatant from recombinant *E. coli* expressing Stx2 and containing lipopolysaccharide (LPS) induces premature delivery of dead fetuses. It has been reported that LPS may combine with Stx2 to facilitate vascular injury, which may in turn lead to an overproduction of nitric oxide (NO). The aim of this study was to evaluate whether NO is involved in the effects of Stx2 on pregnancy. Pregnant rats were i.p. injected with culture supernatant from recombinant *E. coli* containing Stx2 and LPS (sStx2) on day 15 of gestation. In addition, some rats were injected with aminoguanidine (AG), an inducible isoform inhibitor of NO synthase (iNOS), 24 h before and 4 h after sStx2 injection. NO production was measured by NOS activity and iNOS expression by Western blot analysis. A significant increase in NO production and a high iNOS expression was observed in placental tissues from rats injected with sStx2 containing 0.7 ng and 2 ng Stx2/g body weight and killed 12 h after injection. AG caused a significant reduction of sStx2 effects on the feto-maternal unit, but did not prevent premature delivery. Placental tissues from rats treated with AG and sStx2 presented normal histology that was indistinguishable from the controls. Our results reveal that Stx2-induced placental damage and fetus mortality is mediated by an increase in NO production and that AG is able to completely reverse the Stx2 damages in placental tissues, but not to prevent premature delivery, thus suggesting other mechanisms not yet determined could be involved.

## Introduction

Shiga toxin-producing *Escherichia coli* (STEC) cause a significant public health risk due to contamination of food and water supplies. Gastrointestinal infection with STEC causes diarrhea and hemorrhagic colitis, and is the leading cause of hemolytic uremic syndrome (HUS), a systemic complication that is attributed to expression of Shiga toxins (Stx) [1], [2]. HUS is characterized by hemolytic anemia, thrombocytopenia and acute renal failure, and is recognized as the most common cause of acute renal failure in children and the second leading cause of chronic renal failure in Argentina [3], [4]. Argentina has the highest rate of HUS in the world, with a mean of 13.9 cases per 100,000 children younger than 5 years of age [5]. Microorganisms isolated from children with HUS are STEC and produce Stx1 and/or Stx2, with a greater prevalence of Stx2 [6]. Recently, we have demonstrated that the treatment of pregnant rats with a combination of Stx2 and lipopolysacharide (LPS) on day 14–16 of gestation causes maternal lethality in a dose-dependent manner and premature delivery of dead fetuses 1–2 days post-injection [7]. In addition, we observed fetoplacental resorption, placental abruption, intrauterine hemorrhage and fetal death were described [7]. *In vivo* and *in vitro* studies have demonstrated that LPS may combine with Stx to facilitate vascular injury [8], leading to a pathological cascade that involves the production of nitric oxide (NO) [9], [10]. NO has an important role in implantation, decidualization, vasodilation and myometrial relaxation during pregnancy; however, a massive production of NO catalyzed by the inducible form of nitric oxide synthase (iNOS) could cause pregnancy loss [11], [12]. Aminoguanidine (AG), an inhibitor of iNOS activity [13], totally reverses LPS-induced embryonic resorption [12]. These observations led us to investigate whether an up-regulation of NO production in rats could be involved in Stx2-induced preterm delivery of dead fetuses. Our results show that Stx2-induced placental toxicity and fetus mortality is mediated by an increase in NO production.

## Materials and Methods

### Animals

To obtain timed pregnant females, both male and virgin female Sprague-Dawley rats (250–300 g of body weight) from the School of Veterinary Medicine animal facility of the University of Buenos Aires were used.

Mating was performed by placing females in the cages of males for several days. Day 1 of gestation was determined when sperm was observed in the vaginal smear. Animals received food and water *ad libitum* and were housed under controlled conditions of light (12-h light, 12-h dark) and temperature (23–25°C). This study was carried out in strict accordance with the recommendations in the Guide for the Care and Use of Laboratory Animals of the National Institutes of Health. The protocol was approved by the Institutional Committee for the Care and Use of Laboratory Animals of the University of Buenos Aires (CICUAL, Permit Number 1209–10).

### Preparation of sStx2 and dosage of the cytotoxic activity on Vero cells

The stx2 gene was cloned into pGEM-T-Easy (Promega, USA) and recombinant *E. coli* DH5α expressing Stx2 were incubated overnight at 37°C with shaking at 200 rpm in Luria-Bertani broth (Difco Laboratories) supplemented with 100 µg/ml ampicillin (Sigma Aldrich Co. USA). Bacterial cells were then removed by centrifugation, and the resultant supernatant (sStx2) was filtered through 0.22 µm pore size filter units (Millipore Corp, USA) and assayed for toxicity to Vero cells as previously described [14]. The 50% cytotoxic dose (CD_50_) corresponds to the dilution required to kill 50% of Vero cells. Stx2 cytotoxicity in sStx2 was approximately 1×10^4^ CD_50_/ml corresponding to 400 ng/ml when compared with the cytotoxicity of pure Stx2 on Vero cells. Supernatant from *E. coli* DH5α containing only the plasmid was used as control (sControl). Lipopolysaccharide (LPS) content in the culture supernatant was determined using the HEK-Blue LPS Detection Kit (InvivoGen, San Diego, USA). A sample of 1 ml of supernatant contained 30 ng of LPS (75 ng LPS/µg of Stx2 protein).

### Determination of preterm delivery

In order to examine the effects of sStx2 on the delivery time and fetal status, pregnant rats were intraperitoneally (i.p.) injected with 0.05–1.5 ml of sStx2 containing approximately 0.07–2 ng Stx2/g body weight (wt) on day 14–16 of pregnancy (late stage). Control rats were injected with the same volume of sControl. The rats were individually housed under controlled conditions of light, humidity, and temperature, with food and water available *ad libitum*.

Animals were observed every 30 minutes for any signs of morbidity (piloerection, decreased movement), vaginal bleeding, and/or preterm delivery (pups present in the cage) after sStx2 or sControl administration. The beginning of preterm delivery was defined as the delivery of the first pup.

### Experimental design

Pregnant rats on days 15 of gestation were randomly divided into three groups of five rats each. One group was i.p. injected with 0.5 ml of sStx2 containing 0.7 ng Stx2/g body wt, another with 1.5 ml of sStx2 containing 2 ng Stx2/g body wt, and the last one with 0.5 or 1.5 ml of sControl. All rats were anesthetized and killed by cervical dislocation 12 h after treatment, and the kidneys and placentas were removed.

### Determination of NOS activity

The conversion of L-[^14^C]arginine into L-[^14^C]citrulline was used to quantify NOS activity according to a method previously described [15]. Briefly, slices of tissue were weighed, homogenized in a buffer containing 20 mM Hepes (4-(2-hydroxyethyl)-1-piperazineethanesulfonic acid), 25 mM L-valine, 0.45 mM CaCl_2_, and 1 mM DTT (dithiothreitol), and incubated at 37°C with 10 µM L-[^14^C] arginine (0.3 µCi, Amersham Corp, UK) and 0.5 mM NADPH (nicotinamide adenine dinucleotide phosphate). After 15 min, samples were centrifuged for 10 min at 10,000 *g* and applied to a Dowex AG50-X8 column (Na^+^ form, Bio-Rad Lab, USA), and L-[^14^C] citrulline was eluted. L-[^14^C]Citrulline radioactivity was measured by liquid scintillation counting. Enzyme activity is reported as fmoles of L-[^14^C]citrulline per mg of protein per 15 min.

### Western blot analysis

The tissues were homogenized on ice in an Ultra-Turrax homogenizer in a buffer containing 50 mM Tris-HCl, pH 7.4, 10 mM EDTA, 150 mM NaCl, 1% Triton X-100 with 1 µg/ml pepstatin A, 1 µg/ml leupeptin, 5 µg/ml aprotinin, 1 mM phenylmethanesulfonylfluoride, all purchased from Sigma Aldrich Corp (USA). Homogenates were pre-centrifuged at 2,500 *g* for 10 min at 4°C and the collected supernatants were additionally centrifuged at 7,800 *g* for 10 min at 8°C. The supernatants were collected and stored at −70°C until western blotting was performed. Protein concentration was determined with the BCA Protein Assay Kit (Pierce Biotechnology Inc, USA). One hundred micrograms of protein were loaded in each lane. Samples were separated on 7.5% (w/v) sodium dodecyl sulphate-polyacrylamide gel by electrophoresis and transferred to a PVDF membrane (Bio-Rad Lab, USA). The blots were incubated for 72 h at 4°C with the primary antibodies diluted 1:200 in phosphate-buffered saline (PBS). The primary antibody was anti-iNOS rabbit polyclonal (BD Transduction Lab). The membranes were then incubated for 60 min at room temperature with horseradish peroxidase-conjugated goat anti-rabbit IgG antibody (1:3000, Bio-Rad, USA) as secondary antibody. Proteins were detected using the Amersham ECL detection system (Amersham Corp, UK). To determine the uniformity of loading, protein blots were probed with the monoclonal anti-β-actin antibody (1:4000, Sigma-Aldrich Corp, USA). Band intensities were measured using the Quantity One densitometry software package (Bio-Rad Lab, USA). Protein bands were normalized to their respective β-actin bands. The Western blots analysis was carried out with pool materials of 1–2 placentas and 1–2 kidneys from 5 rats corresponding to each experimental condition. The experiments were repeated 4 times.

### Light microscopy

Freshly removed placentas were fixed for 48 h in formol buffer 10% in 0.1 M PBS pH 7.4. The tissue sections were dehydrated and embedded in paraffin. Sections of 5 µm were made by a microtome (Leica RM 2125, Wetzlar, Germany) and mounted on 2% silane-coated slides. The sections were stained with hematoxylin–eosin, and observed by light microscopy (Nikon Eclipse 200, NY, USA).

### iNOS immunohistochemistry

The samples were blocked of endogenous peroxidase with hydrogen peroxide 0.3% in methanol for 10 min and rinsed with PBS. Then, the slides were pre-incubated with non-immune rabbit serum diluted in PBS (1:100) in a humidity chamber at room temperature for 1 h and incubated with anti-iNOS rabbit polyclonal antibody (BD Transduction Lab, USA). The antibody was diluted 1:50 in PBS and incubated in a humidity chamber at 4°C overnight. The immunoperoxidase technique was then performed following the protocol for the RTU Vectastain kit (Vector Corp, UK). The antigen was revealed by diaminobenzidine (DAB, Vector Corp). Finally, the sections were dehydrated, counterstained and mounted for observation.

### Determination of AG effects on Stx2-treated pregnant rats

To study the effects of AG on the Stx2-treated pregnant rats, the following experimental protocol was used. Rats were i.p. injected with 0.5 or 1.5 ml of sStx2 (0.7 ng or 2 ng Stx2/g body wt) on day 15 of gestation. Some of them were treated with 100 µg AG/g body wt 24 h before and 4 h after injection. Some animals were killed 12 h later to evaluate NOS activity and iNOS expression, as well as microscopic alteration of placenta tissues. The other rats were killed at 48 h to observe the fetal status. Gestational sacs were opened and the fetuses and placentas were evaluated macroscopically. The remaining rats were observed up to delivery time. The data were compared with those obtained when the rats were injected with 1.5 ml of sControl.

### Statistical analysis

Statistical analysis was performed using the Graph Pad Prism Software (San Diego, CA, USA). Comparisons between values of different groups were performed using one-way ANOVA. Significance was determined using Tukey's multiple comparison tests for unequal replicates or Student's t-test. Statistical significance was set at P<0.05.

## Results

### Preterm delivery induced by Stx2 is dependent on toxin concentration

**Figure 1:**
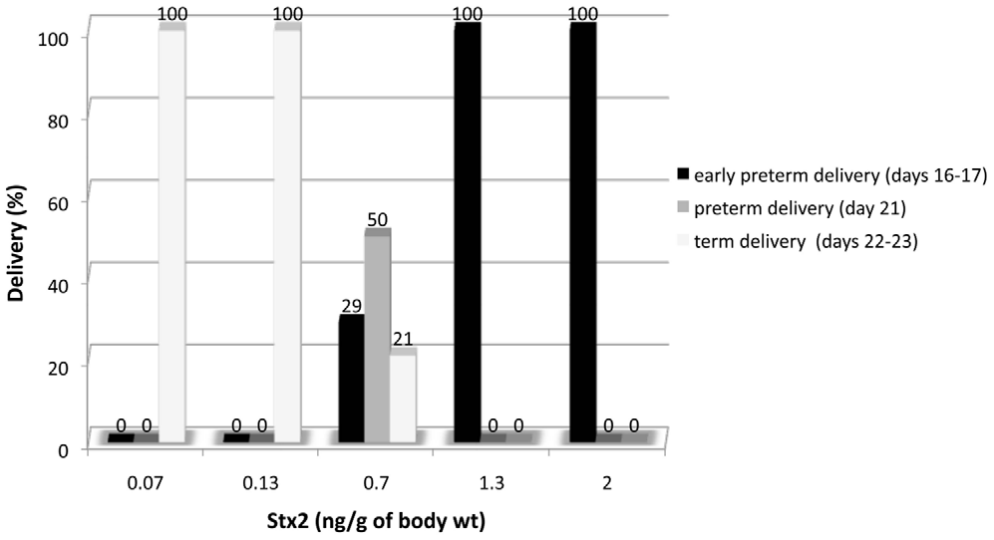
Effects of Stx2 on pregnant rat delivery. Pregnant rats were injected with 0.05–1.5 ml of sStx2 containing 0.07–2 ng Stx2/g body wt on day 14–16 of gestation. Delivery percentages were evaluated up to 12 days post-injection (n = 6–12 rats for each concentration).

Figure 1 shows deliveries of pregnant rats after injection with sStx2 containing different doses of Stx2. All the rats (100%) injected with 1.3 and 2 ng Stx2/g body wt exhibited early premature delivery of dead fetuses (day 16–17 of gestation). In contrast, 100% of rats injected with 0.07 and 0.13 ng Stx2/g body wt delivered live fetuses at term (day 22–23 of gestation). Rats injected with sControl also delivered fetuses at term. It is noteworthy that the injection of 0.7 ng Stx2/g body wt induced preterm delivery of live fetuses on day 21 of gestation in 50% of the Stx2-treated rats, although the neonates did not survive. These results demonstrate that systemic administration of sStx2 causes fetal death in a dose-dependent fashion, without causing maternal lethal effects.

### Effect of sStx2 on NOS activity and iNOS expression

**Figure 2:**
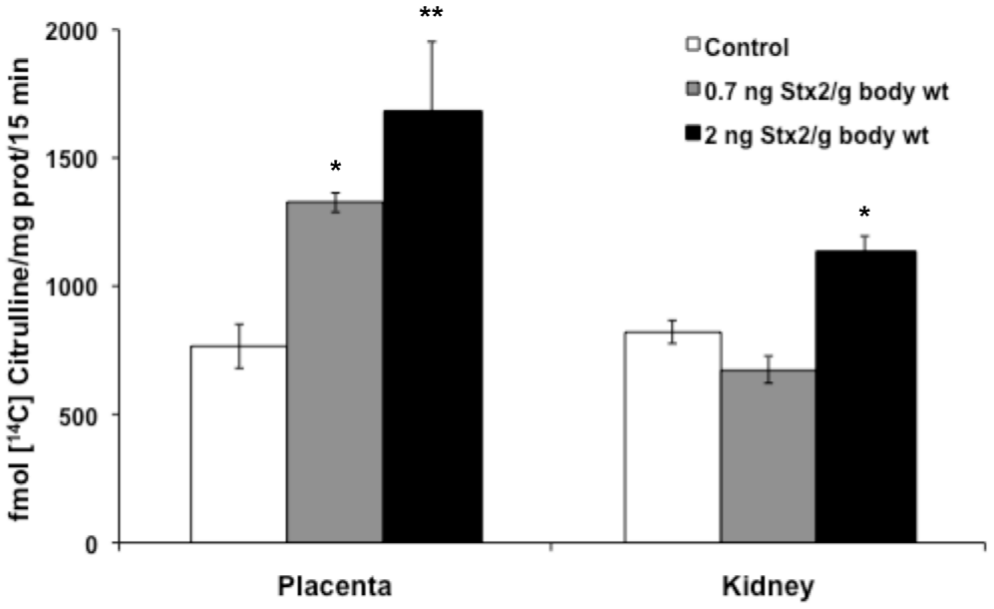
Nitric oxide synthase (NOS) activity in placental and kidney tissues after Stx2 injection. Pregnant rats on day 15 of gestation were injected with 0.5 or 1.5 ml of sStx2 (0.7 or 2 ng Stx2/g body wt, respectively) and killed 12 h post injection. Control rats were injected with the same volume of sControl. NOS activity was quantified by measuring the conversion of L-[^14^C]arginine into L-[^14^C] citrulline. Values corresponding to at least two separate experiments are shown. Values are expressed as the mean ± SEM (n = 6–8). ^*^P<0.05 vs control; ^**^P<0.01 vs control.

To investigate the role of NO in sStx2-induced preterm delivery we analyzed NOS activity by the conversion of L-[^14^C]arginine into L-[^14^C]citrulline. We observed a significant increase in NOS activity in placental tissues of rats injected with sStx2 containing 0.7 ng and 2 ng Stx2/g body wt and killed 12 h after injection (Figure 2). The increase in NOS activity was also observed in kidney tissues of rats injected with 2 ng Stx2/g body wt (Figure 2), although it was not enough to alter the renal functional parameters and the microscopic appearance of kidneys (data not shown).

**Figure 3:**
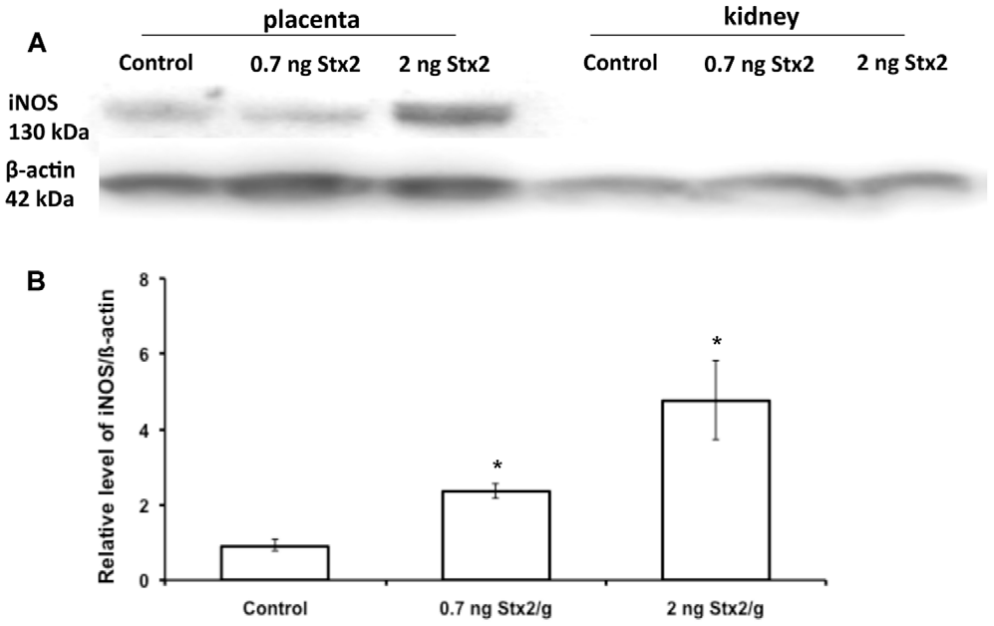
Western blot for iNOS protein expression in placental tissues. Pregnant rats were injected with 0.5 or 1.5 ml of sStx2 (0.7 or 2 ng Stx2/g body wt, respectively) on day 15 of gestation and killed 12 h post injection. Control rats were injected with the same volume of sControl. iNOS expression in placental tissues was detected using a specific polyclonal anti-iNOS antibody. To determine the uniformity of loading, western blots were probed with a monoclonal anti-β-actin antibody. Each value represents a pool of five animals. (A) Representative gel for iNOS expression. (B) Densitometric analysis of the bars. Values correspond to the mean of four different pools of five animals ± SEM. *P<0.01 vs controls.

**Figure 4:**
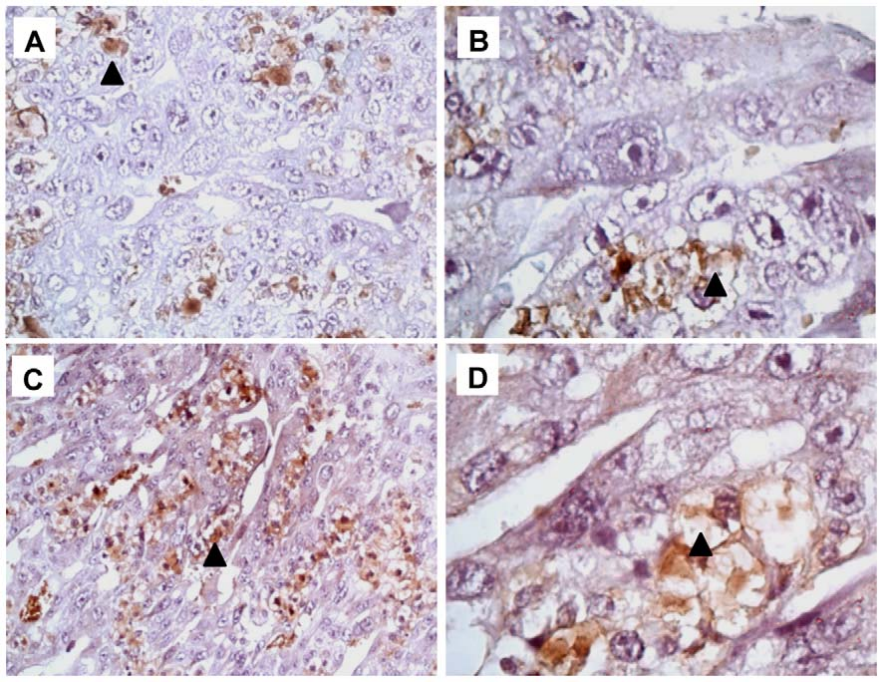
Immunolocalization of iNOS in placental tissues. Pregnant rats were injected with 0.5 ml of sControl or sStx2 (0.7 ng Stx2/g body wt) on day 15 of gestation and killed 12 h post injection. iNOS was detected in trophoblastic giant cells (A, black arrowhead) and glycogen cells (B; black arrowhead) of placentas from control rats. An increased label of iNOS in glycogen cells (C–D, black arrowhead) was observed in placentas from Stx2-treated rats. Original magnification: A and C ×200, B and D ×1000.

Inducible NOS (iNOS) protein was detected as a 130 kDa band by western blot in placental tissues from both control and experimental rats killed 12 h after injection (Figure 3A). The expression of iNOS significantly increased at 0.7 ng Stx2/g body wt and even more at 2 ng (Figure 3B), thus indicating the relationship between toxin doses and NOS activity. iNOS protein was not detected in kidney tissues of rats injected with either sControl or sStx2 (Figure 3B). Therefore, only placental tissues of control and sStx2-treated rats were processed by immunochemistry (Figure 4). In rats treated with sControl, iNOS label was detected in trophoblastic giant cells (Figure 4A; black arrowhead) and glycogen cells (Figure 4B; black arrowhead). Rats treated with 0.7 ng Stx2/g body wt showed an increase in iNOS labeling in glycogen cells (Figure 4C–D, black arrowhead). Similar results were observed using 2 ng Stx2/g body wt (data not shown).

### Effect of AG on NOS activity and iNOS protein expression

**Figure 5:**
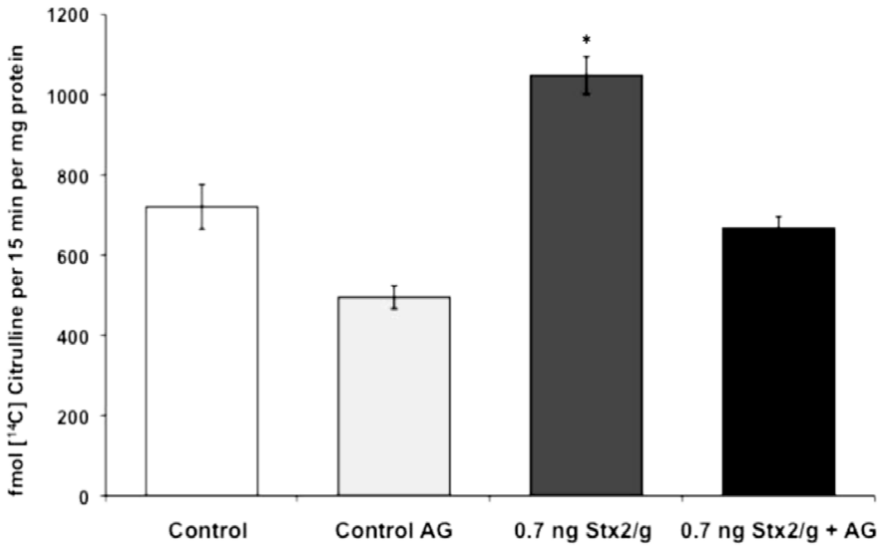
*In vivo* effects of AG on NOS activity in placental tissues. Pregnant rats were injected with 0.5 ml of sStx2 (0.7 ng Stx2/g body wt) on day 15 of gestation and killed 12 h post injection. One group was treated with AG (100 µg/g body wt) 24 h before and 4 h after sStx2 administration. Another was treated only with AG (control AG). Control rats were treated with PBS 24 h before and 4 h after administration of 0.5 ml of sControl. Values are the mean ± SEM (n = 4). ^*^P<0.001 vs other experimental conditions.

The increase in NOS activity detected in placental tissues after injection of sStx2 containing 0.7 ng Stx2/g body wt decreased significantly when pregnant rats were treated with 100 µg AG/g body wt 24 h before and 4 h after Stx2 injection (Figure 5).

**Figure 6:**
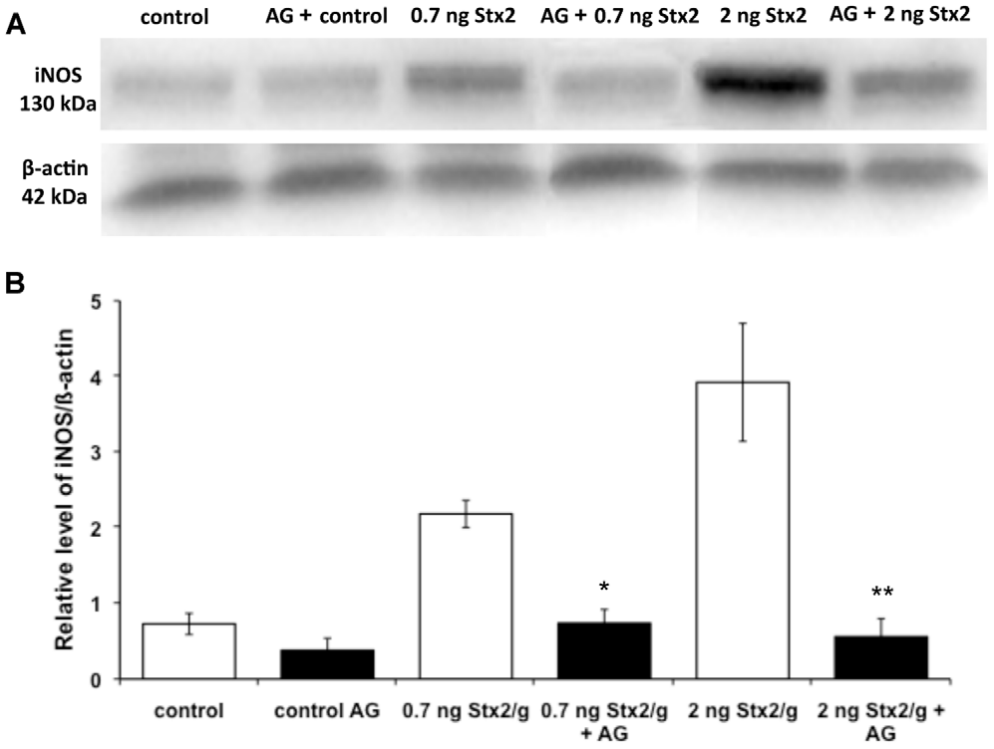
*In vivo* effect of AG on iNOS protein expression in placental tissues. Pregnant rats were injected with either 0.5 ml (0.7 ng Stx2/g body wt) or 1.5 ml of sStx2 (2 ng Stx2/g body wt) on day 15 of gestation and killed 12 h post injection. Some animals of each Stx2 concentration were treated with AG (100 µg/g body wt) 24 h before and 4 h after injection of sStx2, whereas others were treated with AG alone. Control rats were treated with PBS 24 h before and 4 h after injection of sControl. (A) Representative gel for iNOS expression. (B) Densitometric analysis of the bars. Values correspond to the mean of four different pools of five animals ± SEM. ^*^P<0.001 vs 0.7 ng Stx2/g body wt, ^**^P<0.05 vs 2 ng Stx2/g body wt.

The treatment with AG also decreased the iNOS protein expression observed in placentas from rats injected with 0.7 ng or 2 ng Stx2/g body wt. Values returned to the same levels as those observed in placentas from control rats (Figure 6). Both NOS activity and iNOS expression values in placental tissues from AG-treated animals were similar to those observed in controls rats (Figure 6).

### Macroscopic evaluation of the feto-maternal unit after AG treatment

**Figure 7:**
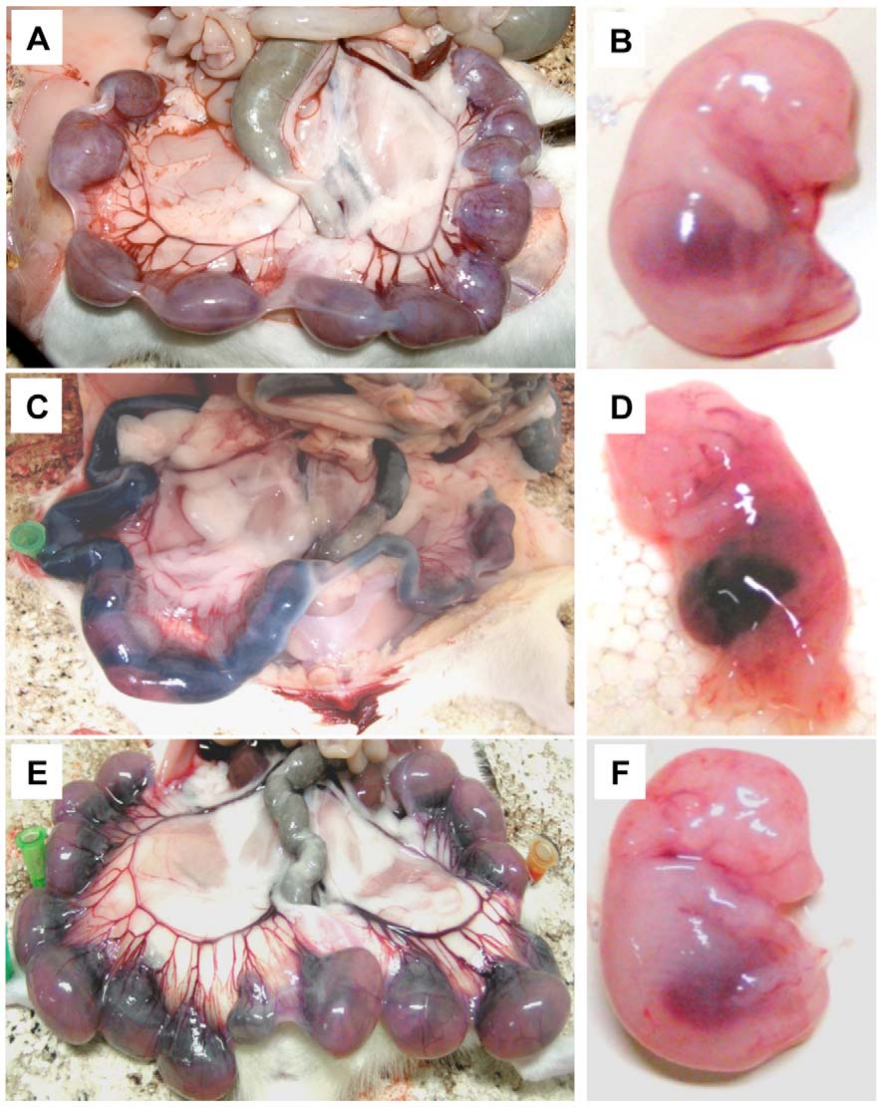
Macroscopic evaluation of pregnant rats treated with AG and sStx2. Uteri and fetuses from pregnant rats injected with 1.5 ml sStx2 containing 2 ng Stx2/g body wt showed intrauterine hemorrhage and fetal death 48 h after sStx2 injection (C, D). The treatment with 100 µg AG/g body wt 24 h before and 4 h after sStx2 injection caused significant reduction of the toxin effects on the feto-maternal unit (E, F). Uteri and fetuses from control rats showed normal fetal growth (A, B).

Uteri and fetuses from pregnant rats injected with sStx2 (2 ng Stx2/g body wt) showed intrauterine hemorrhage and fetal death 48 h post injection (Figure 5C and D). Treatment with 100 µg AG/g body wt 24 h before and 4 h after sStx2 injection caused a significant reduction of Stx2 effects on the feto-maternal unit (Figure 7E and F). Uteri and fetuses from control rats showed normal fetal growth (Figure 7A and B).

### Histology evaluation of placentas after AG treatment

**Figure 8:**
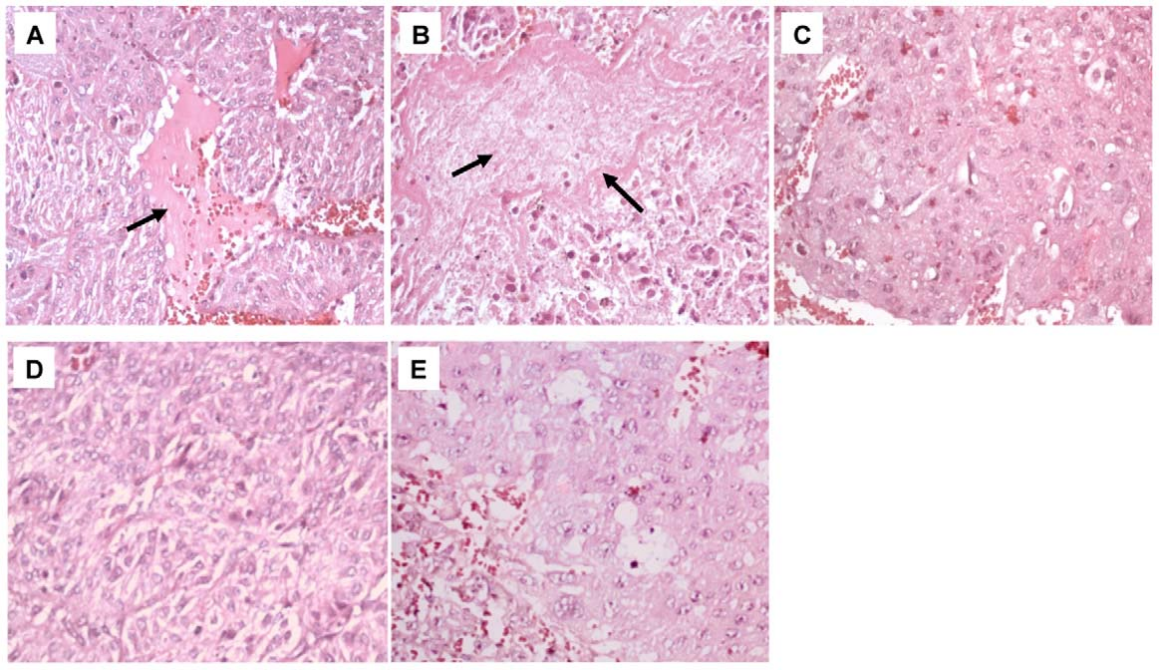
Histology of the placentas from pregnant rats treated with AG and sStx2. Placentas from pregnant rats treated with 0.7 or 2 ng Stx2/g body wt presented necrotic areas (A and B, black arrows) as compared with controls (C). Placentas from rats treated with 100 µg AG/g body wt plus 0.7 (D) or 2 ng (E) Stx2/g body wt did not show histological changes compared with controls (C). H&E. Original magnification: ×200.

Placentas from rats injected with 0.7 or 2 ng Stx2/g body wt and killed 12 h post injection presented necrotic areas (Figure 8A and B, respectively) as compared with those from control animals (Figure 8C). In contrast, the placentas from rats treated with 100 µg AG/g body wt 24 h before and 4 h after injection with either 0.7 or 2 ng Stx2/g body wt (Figure 8D and E, respectively) presented normal morphology indistinguishable from the controls (Figure 8C).

### Effect of AG treatment on the preterm delivery induced by sStx2

Treatment with 100 µg AG/g body wt 24 h before and 4 h after sStx2 injection containing 2 ng Stx2/g body wt did not prevent the premature delivery of dead fetuses. In contrast, all the pregnant rats treated only with AG or sControl delivered normal live pups on days 22 or 23 of gestation. After Stx2-treatment, all rats were able to become pregnant and deliver normal pups at term.

## Discussion

Our data show that sStx2 (a combination of Stx2 and LPS) induces a significant increase in NO production and levels of iNOS protein in placental tissues from pregnant rats, suggesting overproduction of NO plays an important role in sStx2-induced placental toxicity and fetal mortality [7]. Some authors have described high concentrations of NO resulting from the output of iNOS have been described in the pathophysiology of LPS-mediated fetal resorption [12], [16]. These authors demonstrated that LPS causes 100% embryonic resorption in mice, and that this was totally blocked by AG administration 24 h before and 4 h after LPS injection. In our experiments, AG prevented both the overproduction of NO by inhibiting iNOS and the histological damages in placenta observed after sStx2 treatment.

NO is produced from L-arginine under the catalytic control of nitric oxide synthase (NOS). Three NOS isoforms have been identified: the endothelial (eNOS) and neuronal (nNOS) isoforms, which are responsible for basal, i.e. constitutive, NO production, and iNOS, which is activated by a variety of agonists including LPS and tumor necrosis factor (TNF). In contrast to the constitutive NOS isoforms, iNOS is thought to account for the increased NO production seen in pathological states [17].

Although there are several clinical studies on the enhanced NO production in patients with HUS [18], [19], its role in the pathogenesis of the syndrome has not yet been assessed. It has been described that Stx down-regulates eNOS but up-regulates iNOS in cultured human aortic endothelial cells [20]. In contrast, Stx reduces both eNOS and iNOS in human glomerular endothelial and mesangial cells [21]. In a baboon model of Stx-mediated HUS [22], NO production (measured as urinary NO) increased during the initial 12 hours after Stx1 injection and then decreased below baseline values. In an HUS mouse model obtained by Stx2-injection, NO overproduction was attributed to endothelial injury and/or the inflammatory response associated with iNOS activity. However NOS inhibition caused an increase in Stx2-mediated renal damages and lethality [23]. In our experiments, the lack of iNOS expression in renal tissues of sStx2-treated pregnant rats in combination with a normal renal physiology and histology indicates the inability of Stx2 to affect the mother's kidneys. The complete (100%) maternal survival at the sStx2 doses used also show that the brain was not affected. The fact that Stx2 susceptibility in the kidney and brain is lower than in the placenta could be the consequence of a high blood flow distribution to the feto-placental unit. Since Stx2 may be captured in the placental and embryonic tissues, its availability (concentration) in blood necessary to cause the effects on other target organs described in non-pregnant rats treated with similar sStx2 doses is decreased [24].

Here we found that AG did not prevent premature delivery, thus indicating that other mechanisms not yet determined could be involved. It is well known that Stx may act in concert with LPS to elicit cellular dysfunction [25]. Stx can induce the production of tumor necrosis factor (TNF) and interleukins-1 (IL-1) and –6 (IL-6), by macrophages rendering the endothelial cells more sensitive to the toxin [26], [27]. It is possible that cytokine stimulation caused by sStx2 may be responsible for premature delivery. Many studies conducted in human and experimental animals have established that a correct balance of cytokines at the maternal-fetal interface is an essential requirement for proper placental development and, therefore, reproductive success [28], [29]. Additionally, there is evidence supporting key roles of prostaglandins (PGs) [30], [31]. Changes in uterine PG production appear to be involved in the regulation of myometrial activity during pregnancy and labor. Thus, further studies about cytokines and PG induction in the placenta, uterus and fetus will be necessary to address whether these pro-inflammatory mediators are involved in the premature delivery observed in response to Stx2. Finally, we cannot discard that NO could react with the superoxide radical and generate peroxynitrite, a key oxidant and nitrating molecule, which could initiate toxic reactions by introducing tyrosine nitration [32].

In summary, the present results allow us to conclude that the Stx2-induced premature delivery in rats in the late gestational stage is, at least in part, mediated by an increase in NO production resulting from the output of iNOS.
